# Chloroplasts with clefts and holes: a reassessment of the chloroplast shape using 3D FE-SEM cellular reconstruction of two species of *Chlamydomonas*

**DOI:** 10.1007/s00709-024-01990-7

**Published:** 2024-09-23

**Authors:** Naoki Sato, Mayuko Sato, Mayumi Wakazaki, Takashi Moriyama, Takashi Hirashima, Kiminori Toyooka

**Affiliations:** 1 Graduate School of Arts and Sciences, The University of Tokyo, Komaba, Meguro-Ku, Tokyo 153-8902 Japan; 2 RIKEN Center for Sustainable Resource Science, Yokohama, Kanagawa 230-0045 Japan; 3 Present Address: Kyoto Research Laboratory, Beacle Inc, Kyoto, 606-8305 Japan; 4 Present Address: Faculty of Life Sciences, Kyoto Sangyo University, Kyoto, 603-8555 Japan

**Keywords:** Array tomography, *Chlamydomonas*, Chloroplast shape, Three-dimensional reconstruction, Lipid droplet topology

## Abstract

Chloroplasts are usually considered spheroid organelles, but this is not the only shape of chloroplasts. The chloroplast of *Chlamydomonas* has been typically described as cup-shaped. However, in old studies, it was also modeled as a complex shape with “perforations” or windows. Here, we reconstructed the cellular architecture of *Chlamydomonas reinhardtii* and *C. applanata* using an array tomography system installed on a field emission scanning electron microscope. *C. reinhardtii* chloroplasts resembled a baseball glove or a cup without a side, featuring numerous large and small holes that may facilitate the transport of metabolites and proteins produced in the Golgi apparatus fitted in the holes. In a lipid-accumulating, high-light condition, the chloroplast volume increased by filling the side cleft with an entire wall. Many accumulated large lipid droplets were accommodated within the chloroplast holes, which could have been considered as “chloroplast lipid droplets.” Mitochondrial meshworks surrounded the chloroplast. *C. applanata* chloroplasts appeared like a folded starfish or a cup with many side clefts and a few holes. There was a single mitochondrion or two that branched in a complex form. Tight contacts of various organelles were also found in *C. applanata*. These reconstructions illustrate the complexity of chloroplast shape, which necessitates a revised understanding of the localization of lipid droplets and the evolution of chloroplasts: The prevailing image of the spheroid chloroplasts that reminds us of the similarity between chloroplasts and cyanobacteria is no longer tenable.

**Supplementary Information:**

The online version contains supplementary material available at 10.1007/s00709-024-01990-7.

## Introduction

Chloroplasts are usually described as spheroid organelles, about 5 µm in length and 1–2 µm in width. This is a typical view of mesophyll chloroplasts of model plants and could provide a similarity of chloroplasts and cyanobacteria in the early days of endosymbiotic theories (Mereschkowsky 1905; Schimper 1883). The image of chloroplasts remained unchanged over a century. However, discovering holes and pockets (invaginations) in plant chloroplasts (Oi et al. 2017; Yamane et al. 2018) by three-dimensional (3D) reconstruction from serial sections opened a new view of the morphology of chloroplasts. Subcellular structures of phytoplankton have been elucidated by 3D imaging based on focused ion beam (FIB)-SEM (Uwizeye et al. 2021). The 3D structure of a *Chlorella pyrenoidosa* cell was related to growth conditions (Feng et al. 2023).

The 3D structure of *Chlamydomonas reinhardtii*, a model microalga, was studied in the 1970s using TEM with serial sections, which revealed large “perforations” (windows) and small holes in the chloroplast (Schötz 1972; Schötz et al. 1972). These papers presented monochromatic photographs of the wooden assembly of cell components, and the exact structure is no longer available to readers. The current sourcebook (Harris 2009) on Chlamydomonas (the common name for *C. reinhardtii* is spelled in the Roman face) describes the complexity of chloroplast structure by citing the figures of Schötz on page 28 (especially Fig. 2.2) and page 57, which was unfortunately hardly understandable. However, the same book also wrote “a cup-shaped chloroplast” in some illustrations, such as Fig. 1.1 on page 2 and Fig. 2.18 on page 58. Unfortunately, this could be easier for many readers, so even the Chlamydomonas researchers believe that the Chlamydomonas chloroplast is “cup-shaped.” A recent study of 3D reconstruction of Chlamydomonas cells by X-ray tomography showed the chloroplasts as just a spherical object without holes or windows (Hummel et al. 2012). These authors cited many old ultrastructural studies but did not cite the papers by Schötz. The paper reporting the 3D structure of Chlamydomonas chloroplast using cryo-electron tomography did not show the overall structure of the chloroplast and described nothing about the chloroplast morphology (Engel et al. 2015; Wietrzynski et al. 2020). The chloroplast holes have been intentionally or unintentionally disregarded in most literature on Chlamydomonas. Only Uniacke and Zerges (2007) mentioned the chloroplast structure in the text: “a few elongated finger-like lobes … large perforations in the resulting rim” (p. 3641, right column) also citing Gaffal et al. (1995) on the 3D reconstruction based on serial section of TEM. This epistemological gap could be because the original figures showing the 3D structures (Schötz 1972, reproduced as Fig. 2.2 in Harris 2009) were hard to understand, and the papers were written in German.

During the study on the lipids of a distantly related green alga, *C. applanata* (Hirashima et al. 2017), we noted that it has a different cell organization: the nucleus is located at the posterior end of the cell, distal from the flagella. In other words, the chloroplast might not be “cup-shaped.” We chose this alga to compare the chloroplast structure and the entire cell architecture with *C. reinhardtii*. Visviki (2000) described the ultrastructure of *C. applanata* UTEX 230, which was a different strain from the one used in the present study.

In this study, we reconstructed the 3D architecture of *C. reinhardtii* and *C. applanata* cells to show the complex structures of the chloroplast using field emission scanning electron microscope (FE-SEM) equipped with auto-capture for array tomography (ACAT, or simply serial section SEM) system. Additionally, we showed the spatial arrangement of various cell organelles regarding the chloroplast, thus enabling the understanding of enigmatic chloroplast lipid droplets. A topological reflection on the 3D structure of chloroplasts is also exposed for future development of the structural study. Finally, chloroplast morphology is discussed in the context of the evolutionary relationship between chloroplasts and cyanobacteria.

## Materials and methods

### Growth of organisms

*Chlamydomonas reinhardtii* P.A.Dangeard CC-1010 was grown in the Modified Bristol’s Medium (MBM) (Watanabe 1960) at 25°C. In the standard condition, the culture was aerated with 1% CO_2_ with fluorescent lamp illumination at 40 µE m^−2^ s^−1^. In the lipid-accumulating, high-light condition, the culture was aerated with 2% CO_2_ with light at 200 µE m^−2^ s^−1^. The cells were harvested seven hours after the transfer to this particular high light condition (Moriyama et al. 2018). *C. applanata* NIES 2202 Pringsheim was grown in the standard condition for *C. reinhardtii* (Hirashima et al. 2017). Note that this strain of *C. applanata* was initially described as *C. aggregata* (Deason and Bold 1960), but included in *C. applanata* according to the taxonomical revision by Ettl and Schlösser (1992).

### Sample preparation

The cells were harvested by centrifugation and re-suspended in 0.1 M sodium cacodylate buffer (pH 7.2). They were fixed with 1% glutaraldehyde for two hours at room temperature and, after washing in cacodylate buffer, post-fixed with 1% osmium tetroxide for 2 h on ice. The cells were then embedded in agar blocks for further dehydration in ethanol series and propylene oxide. Finally, they were embedded in the Quetol 812 resin (Nisshin EM, Tokyo).

### Thin sections and microscopy

Serial ultrathin sections (100 nm thick) were cut using a diamond knife (Ultra 35°, Diatome, Nidau) on an ultramicrotome (Leica EM UC7, Leica Microsystems, Wetzlar) and placed on plastic plates or silicon wafers. The sections were stained with 0.4% uranyl acetate followed by lead stain solution (Merck (Sigma-Aldrich), Darmstadt) and coated with osmium tetroxide by an osmium coater (HPC-1SW, Vacuum Device, Ibaraki). Images of serial ultrathin sections were captured with a FE-SEM (Regulus8240; Hitachi High-Tech, Tokyo) equipped with ACAT and a low-angle backscattered electron (LA-BSE) detector at an accelerating voltage of 2 kV.

### Image processing

**Fig. 1:**
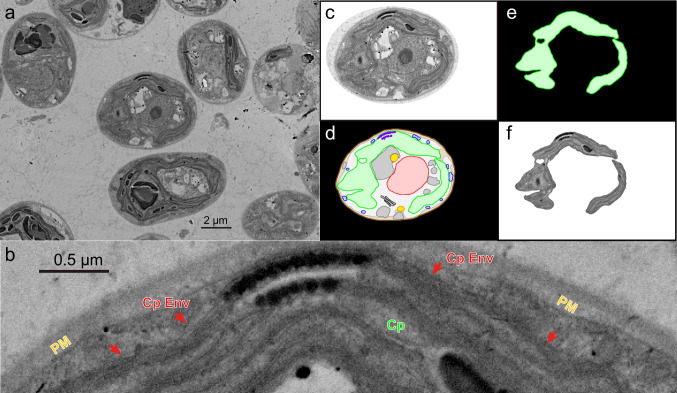
Sample image of chloroplast envelope and the flow of image processing. **a** A FE-SEM image of *C. reinhardtii* cells grown in the standard condition. **b** Enlargement of a part of the cell in the center of panel **a**. Cp, chloroplast; CpEnv (with a red arrow), chloroplast envelope membranes; PM, plasma membrane. **c–f** The flow of image processing. **c** Cropped image of the central cell in panel **a**. **d** The same cell with organelles delineated and colored (“color-painted organelles”). **e** Isolated chloroplast drawing. **f** Clipped chloroplast SEM image using the drawing in **e** as a mask. The clipped chloroplast SEM image was used for 3D reconstruction by the Fluorender software, whereas the color-painted chloroplast drawings (namely, without internal structures) were used for 3D reconstruction by the 3D Slicer software

A single cell was selected in the images of serial sections, and all serial images of the cell were manually aligned and trimmed using Adobe Photoshop version CS6 and ImageJ version 1.45. This step, often called registration, could be performed automatically by 3D image processing software, but manual registration was better for aligning a single cell. For each image, the outlines of various cellular components, such as cell wall, plasma membrane, chloroplast, nucleus, lipid droplet, mitochondrion, Golgi apparatus, eye spot, flagellar 9 + 2 microtubule bundle (plus basal body), and vacuole, were traced manually (namely, manual segmentation) in individual layers using Adobe Illustrator CS6 (Fig. 1). We found that the advantage of FE-SEM over TEM is that the membranes are cleanly delineated. An obliquely cut membrane gives a vague trace in TEM (see explanation in Moriyama et al. 2018), but FE-SEM gives a surface view with a precise contour. See, for example, Fig. 1b, showing the chloroplast envelope in its entire length. The traces of each component were combined and stored as a single multi-TIF image file using the ImageJ software.

### Three-dimensional reconstruction

For 3D image viewing, the biomedical software 3D Slicer version 4.10.2 and 4.11.20210226 (https://www.slicer.org/), and the fluorescence image manipulating software Fluorender version 2.24 and 2.26.3 (https://www.sci.utah.edu/) were used. The 3D Slicer software constructed a three-dimensional surface model for each object using the “Grayscale Model Maker” module and smoothed using the “Surface Toolbox.” The final surface models were stored as STL files. A simplified version of the STL models for distribution was prepared by re-meshing the original STL models with the “Surface Toolbox” of 3D Slicer version 5.4.0. Volumetric analysis was performed using the module “Segment Statistics.” For the simplicity of manipulation, the models were constructed with a nominal scaling factor of 10^5^; namely, an object of 1 µm is represented as 100 mm in the model.

### Model presentation

For graphic presentation, the STL models of cellular components were arranged and manipulated by Shade 3D software version 16 (Forum8, Tokyo). The dimensions of chloroplast STL model were measured according to the “information” command of Shade 3D.

## Results

### Three-dimensional structure of *C. reinhardtii* cells grown in the standard condition

First, we must emphasize that the major interest in the present study is the chloroplast shape. However, during the reconstruction of the 3D chloroplast structure, we also obtained data on the other cell components as well as the cell shape, which are also useful information to researchers. Therefore, we will describe the cell structure in detail in the Results section, but the main focus of the study remains the chloroplast throughout this study.

**Fig. 2:**
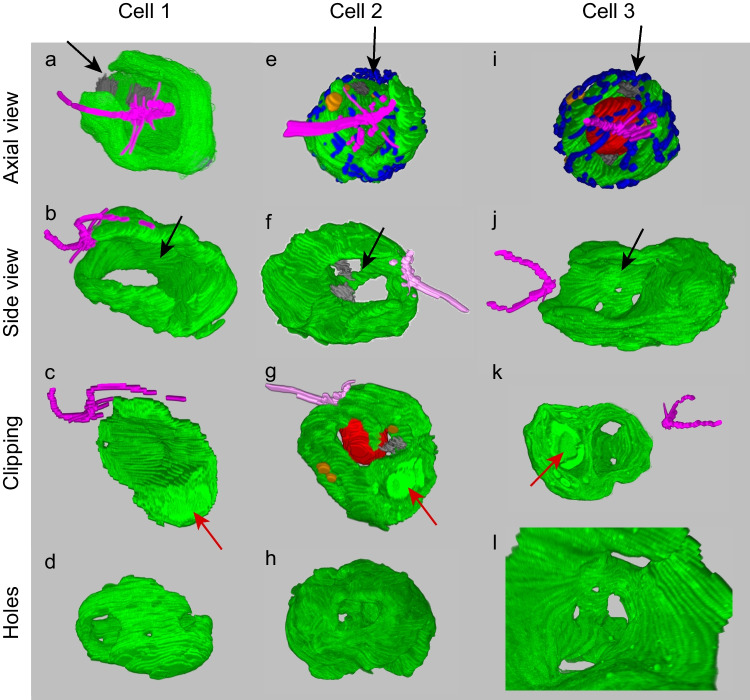
Different views of the *C. reinhardtii* chloroplasts of three cells (Cell 1, Cell 2, and Cell 3) grown in the standard condition. The 3D views were reconstructed from the multi-TIF files for the clipped chloroplast SEM images and the color-painted drawings of the other organelles with the Fluorender software. The structures other than chloroplast are not shown in all panels. All the images show perspective views with similar scaling except in panel (**l**), which is an enlarged image to show small holes. Top (**a, e, i**), anterior view, namely, the view from the flagella side. The black arrow indicates the large opening of the chloroplast. Second row (**b, f, j**), side view from the large opening showing the holes in the opposite chloroplast lobe. Third row (**c, g, k**), view with clipping to show the pyrenoids (red arrows). Bottom (**d, h, l**), view from the chloroplast lobe to show small holes. Green, chloroplast; purple, flagella; red, nucleus; blue, mitochondrion; orange, lipid droplet; dark gray, Golgi apparatus. Cell wall, plasma membrane, and vacuoles are not included in the figures to show the internal structure of the cell

Figure 2 shows the 3D structures of the chloroplast of three *C. reinhardtii* cells grown in the standard condition, viewed from different angles. Each cell has a single chloroplast and two flagella. As a marker of the orientation of chloroplast within the cell, flagella with the basal body (purple) are shown with the chloroplast (green). We define the base of the flagella as the anterior end of the cell (and chloroplast) because the cell swims with the flagella forward. The nucleus (red), Golgi apparatus (dark gray), mitochondrial meshwork (blue), and lipid droplet (orange) are also added in some images to give an idea of the cell structure. However, the organelles other than chloroplasts and flagella are hidden to show the complex structures of the chloroplast in most figures. The figures (including those that follow) do not explicitly show vacuoles, cell walls, and plasma membranes, although they were also reconstructed. The structures are clipped to show the pyrenoid within the chloroplast at the posterior end (arrow in red) in Fig. 2c, g, k. Obviously, the chloroplasts were not simply cup-shaped. The posterior end of each chloroplast was a thick bottom part containing the pyrenoid. A wall or lobe (lined by the internal thylakoid membranes that are identified in the original SEM images) extended from the bottom part toward the anterior end, but a large opening or cleft was found on the flank. There were many holes in the wall part of the chloroplast. Some of them were as large as several micrometers (about 1/3 of cell length) in diameter (Fig. 2b, f, j, arrow in black). The chloroplast was like a baseball glove catching the nucleus, having an opening on one side. Because all the figures and movies are presented in the perspective view, the size of the objects was hardly shown by a scale bar. Instead, the dimensions of the chloroplasts are presented in Supplementary Table S1 (Online Resource 1). Supplementary Movie S1 (Online Resource 2) shows the 3D structure of the chloroplast of one of the three cells (Cell 2) from various points of view. Lipid droplets and Golgi apparatuses are also shown. Each cell had two Golgi apparatuses, each located either within the large opening of the chloroplast on the flank or near a large hole in the chloroplast wall on the opposite side. This arrangement seemed favorable for transporting Golgi-processed products toward the cell surface for further export. Some lipid droplets were also located within the small holes of the chloroplast.

Supplementary Movie S2 (Online resource 3) shows the rotating view of the chloroplast of the three cells in Fig. 2. The three chloroplasts were different in detail, but the general forms were similar. Each chloroplast consisted of the basal part, including a pyrenoid, and the lobe or flat wall. The wall extended from the basal part to cover the nucleus and the interior cytoplasm but did not completely wrap them. A large opening or cleft was found from the basal part to the anterior end. This opening corresponded to a side of the basal body; the flagella were orthogonal to the direction of the opening. The chloroplast cleft was oblique to the cell axis, and this helical turn was similar in the three chloroplasts. This helicity could be related to the helical cell motion: the cell rotates when swimming and moves along a helical trajectory (See, for example, Sato et al. 2018).

Supplementary Figure S1 (Online Resource 1) shows the details of Cell 2 focusing on mitochondria, nucleus, and pyrenoid. Mitochondria formed virtually a single meshwork surrounding the cell. Some parts of the meshwork appeared missing, but this could be because we only traced the surface of each section: the thin thread of mitochondrial meshwork became discontinuous from a section surface to another. This is a disadvantage of FE-SEM in 3D reconstruction in contrast to TEM, which gives an overlapped, vague image of an object seen through the section that becomes continuous from an image to the next. The nucleus was located in the middle of the space formed by the baseball glove-like chloroplast. The anterior end of the nucleus is close to the basal body, which was modeled as a single object with flagella (purple).

These observations clearly indicate that the chloroplast itself has a large cleft and numerous holes of various sizes. This finding is essentially consistent with the older figures presented by Schötz (1972). In other words, holes are already present in the chloroplast even before they accumulate a large amount of lipid droplets.

### Three-dimensional structure of *C. reinhardtii* cells grown in the lipid-accumulating condition

**Fig. 3:**
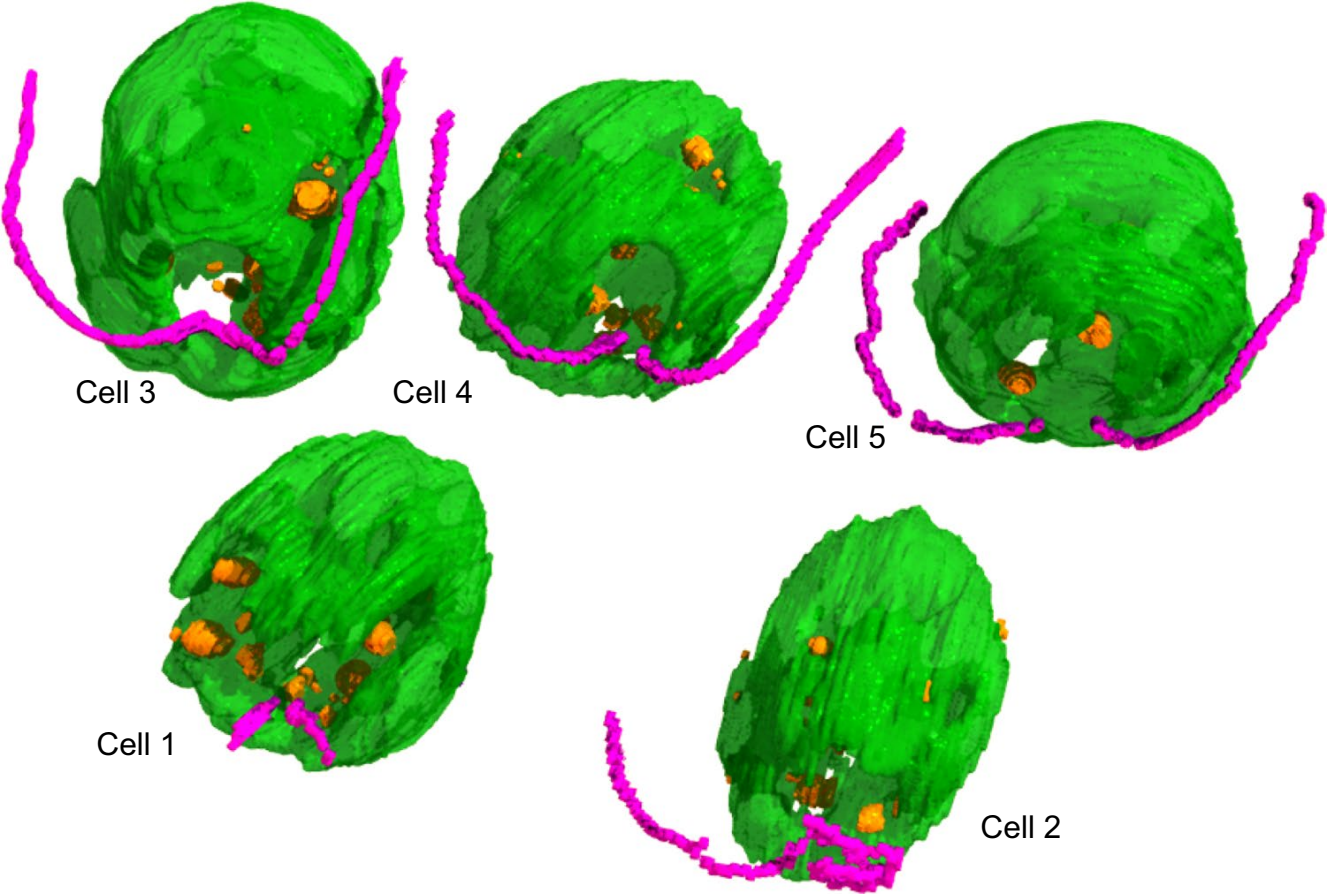
3D structures of the *C. reinhardtii* cells grown in the lipid-accumulating condition. The 3D surface models created by the 3D Slicer software were assembled using the Shade 3D software. Not all parts of flagella were modeled. For color codes, see the legend for Fig. 2

Then, we analyzed the cells grown under the particular high-light condition we used in a previous study (Moriyama et al. 2018), in which lipid droplets rapidly accumulated, and another study (Goold et al. 2016) had suspected their eventual localization within the chloroplast in a similar condition. Figure 3 shows the 3D structure of chloroplast in the cells grown in the lipid-accumulating condition. A rotating view of the same 3D structure is presented in Supplementary Movie S3 (Online Resource 4). Lipid droplets are also shown in both presentation materials. In this condition, the chloroplast was more like a cup but with many holes in the wall. The lobe wall part of the chloroplast wrapped almost perfectly the nucleus (not shown) and its surrounding cytoplasm with a small opening. However, there were many small holes in the lobe wall. Many lipid droplets were found within the small holes in the chloroplast lobe, whereas we never observed lipid droplets inside the chloroplasts, as in the standard growth condition. Supplementary Fig. S2 (Online Resource 1) shows four representative lipid droplets in serial sections. The image series show that LD1 and LD4 were found in a chloroplast opening. LD3 was a lipid droplet closely contacting a vacuole, whereas LD2 was a lipid droplet surrounded by a vacuole. Localization of some lipid droplets within the chloroplast openings and tight contact of some other lipid droplets with vacuoles were new findings in 3D reconstruction with serial sections.

Supplementary Fig. S3 (Online Resource 1) presents more intuitive images showing 3D and cross-section views of some lipid droplets in the chloroplast holes. The 3D views were created by the 3D Slicer software using the STL structure data. The cross-section views were obtained by the Fluorender software using the stacked images. LD1 and LD4 in serial sections are viewed with a more realistic impression. This way, some of the large lipid droplets are fitted in the chloroplast holes and viewed as “chloroplast lipid droplets,” depending on observation methods.

**Fig. 4:**
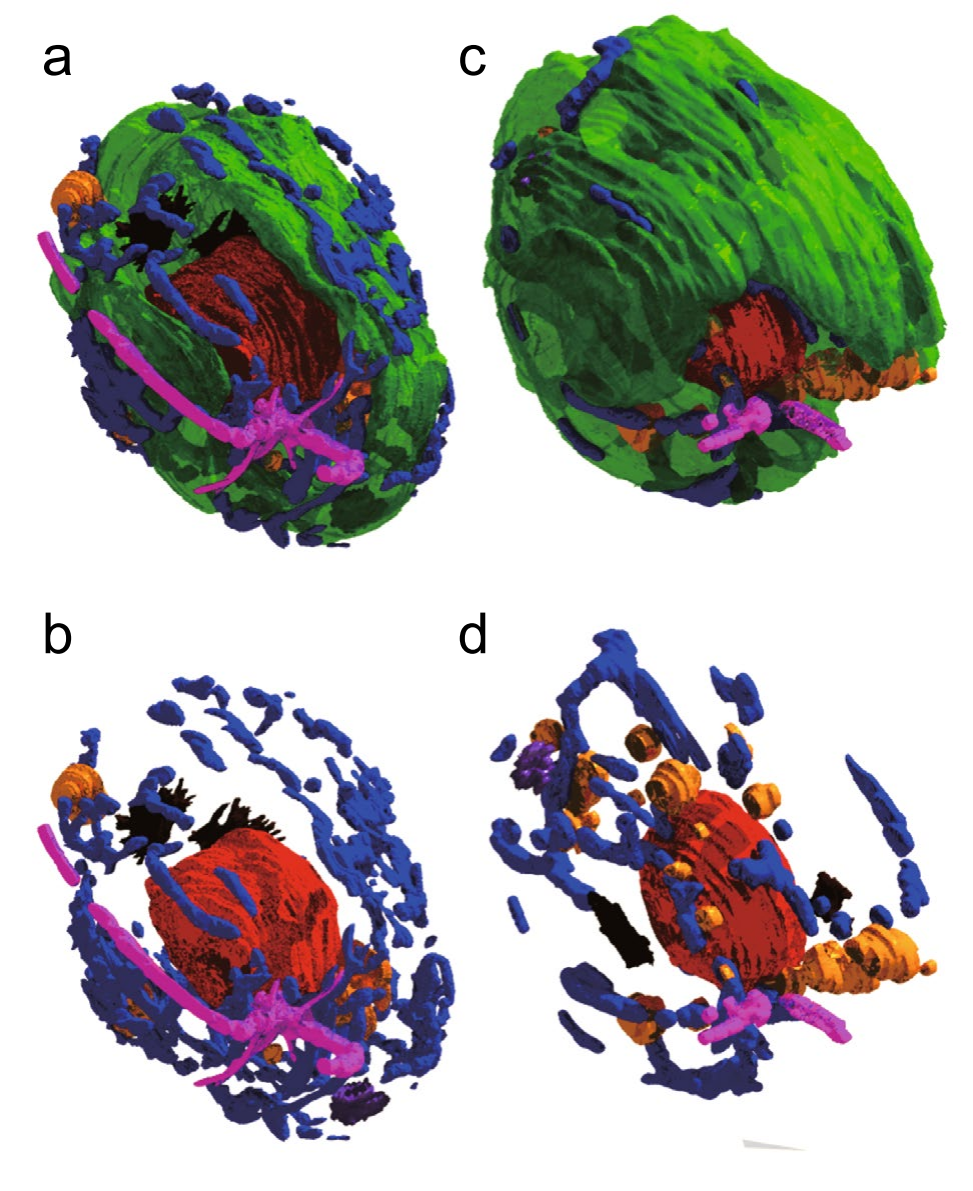
Comparison of the *C. reinhardtii* cells grown in the standard (left) and lipid-accumulating (right) conditions. Top (**a, c**), whole cell view without cell wall, plasma membrane and vacuoles. Bottom (**b, d**), view without chloroplast. For color codes, see the legend for Fig. 2

A comparison of chloroplasts in the two growth conditions is presented in Fig. 4 with other organelles. Views with or without chloroplast are provided to show the 3D arrangement of other organelles. The chloroplast-surrounded structure’s overall size was larger in the lipid-accumulating condition (Fig. 4c and d). Precise measurements of various cell parts will be described in the next section. The opening of the chloroplast was fairly reduced in the lipid-accumulating cell. The arrangement of the mitochondrial meshwork seemed to differ in the two cells, but we cannot identify the structural difference in exact words at this resolution. As expected, lipid droplets were more abundant in the lipid-accumulating cell. In the standard condition, the Golgi apparatuses were located in the cytoplasm between the nucleus and the chloroplast bottom part. In contrast, they were present on both sides of the nucleus in the lipid-accumulating condition. In both cases, they were close to the opening or the hole of the chloroplast, which is considered to be favorable for metabolite traffic, as mentioned above. A rotating view of the two chloroplasts with pyrenoid and eye spot is also shown in Supplementary Movie S4 (Online Resource 5, left and center in the starting view). In this movie, the chloroplasts were made translucent to show the objects in the interior. The pyrenoid was located within the basal part of the chloroplast, but it was more prominent in the lipid-accumulating condition. In both cells, the eye spot was located in the chloroplast lobe in the middle of the cell, at 45° clockwise from the right flagellum if seen from the anterior end, with the chloroplast opening up.

### Volumetric analysis of cell components

**Fig. 5:**
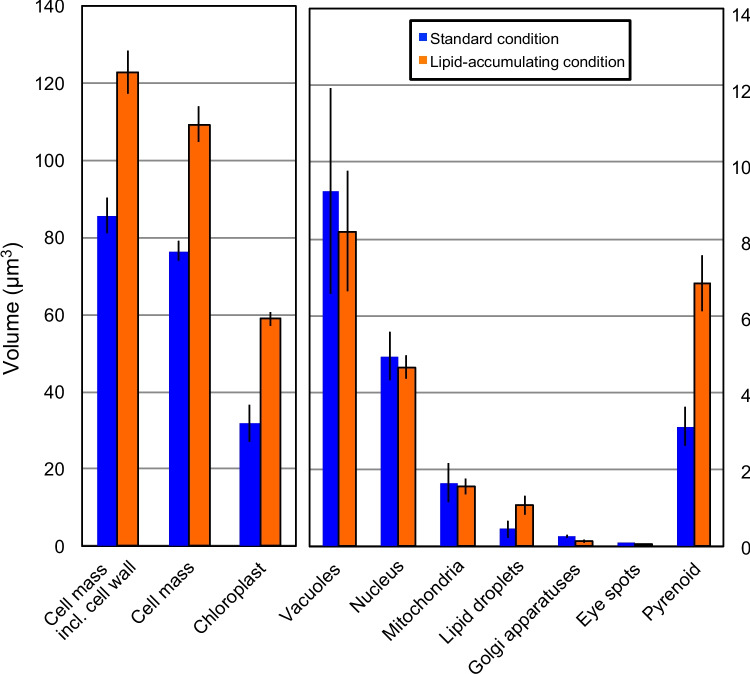
Comparison of the volumes of various cellular parts of *C. reinhardtii* grown in the standard (blue) and lipid-accumulating (orange) conditions. The volumetry was based on the STL model constructed by the 3D Slicer software. Each error bar indicates the standard deviation (*n* = 3 for the standard condition, *n* = 5 for the lipid-accumulating condition)

Volumetry of various cell components and the entire cell of *C. reinhardtii* was performed using the 3D Slicer tool (Fig. 5). The chloroplast size was doubled in the lipid-accumulating condition, and the pyrenoid size was also doubled accordingly. The cell mass, either with or without the cell wall, differed due to the difference in chloroplast size. As noted above, the total volume of lipid droplets was more than doubled in the lipid-accumulating condition. The size of the nucleus, the vacuoles, and the mitochondria did not significantly differ between the two growth conditions.

### Three-dimensional structure of *C. applanata* cells

**Fig. 6:**
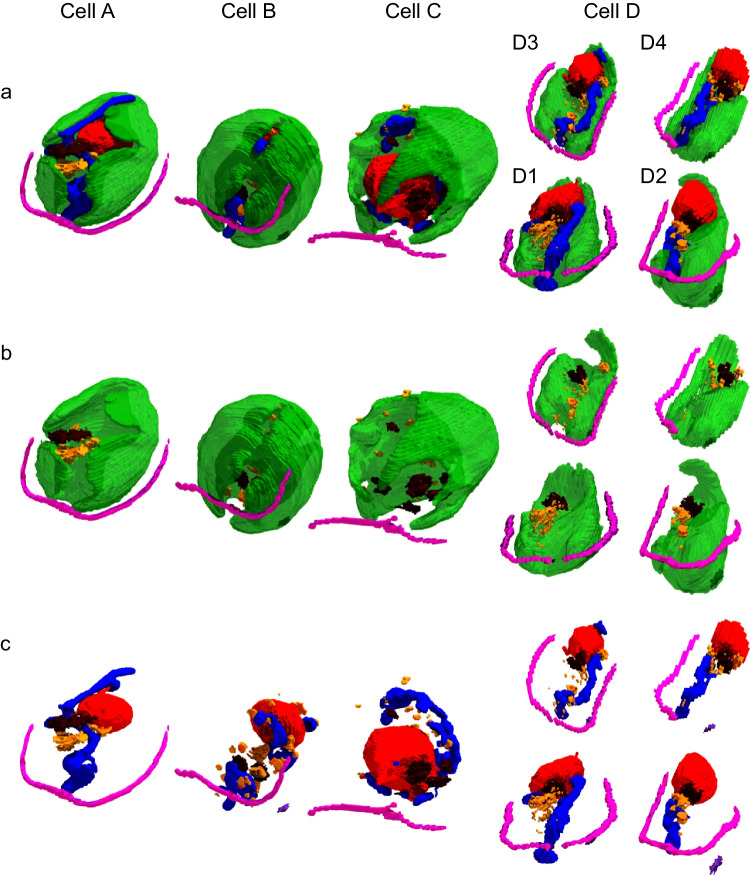
3D structures of *C. applanata* cells. Four cells are arranged according to putative progress of cell cycle. **a** Whole cell view without cell wall, plasma membrane, and vacuoles. **b** View of chloroplast and flagella with lipid droplets and Golgi apparatuses. **c** View as **a** but without chloroplast. Cell D consisted of four daughter cells, D1, D2, D3, and D4. Dark purple, eye spot. For other color codes, see the legend for Fig. 2

Figure 6 shows the 3D structure of *C. applanata* cells. Cells A and B were more or less typical, as the nucleus was at the posterior end. A rotating view of the cells is shown in Supplementary Movie S5 (Online Resource 6). Cell A was an elongated cell having a chloroplast with a large opening spanning from the anterior to the posterior. Additional clefts were also found in the chloroplast. The pyrenoid was located in the middle of the chloroplast, opposite the opening's center (see Supplementary Movie S4 in Online Resource 5, right). The nucleus was located at the posterior end. Curiously, two protrusions of the nucleus (only one of them is visible in Fig. 6c) invaginated into the single, large mitochondrion with complex branches located in the middle of the cytoplasm. A single, large Golgi apparatus was found at the anterior side of the nucleus. Lipid droplets were also found in the cytoplasm. No eye spot was detected in any section of this particular cell, although even a single eye spot is usually observed in several contiguous sections.

Cell B was more rounded than Cell A, but a large opening was found in the chloroplast, encompassing nearly the entire chloroplast. An eye spot was found within the chloroplast, 45° to the flagella. A pyrenoid was located in the middle of the chloroplast rather than at the posterior end.

Cell C was very different, and maybe before cell division (see below), because its size was large, the nucleus was located at the anterior end, and there were four or more large and small Golgi apparatuses. No eye spot was detected. Numerous starch granules filled the chloroplast, whereas pyrenoid was not identified.

Cells D1, D2, D3, and D4 were daughter cells encapsulated within the mother cell wall. We call the set of these daughter cells “Cell D” here. Interestingly, each chloroplast of the daughter cells was flattened, just like a spatula, similar to the single lobe of the entire chloroplast found in Cell C. Nevertheless, each chloroplast contained a pyrenoid at its center. Each daughter cell had an eye spot, located 45° to the flagella, two Golgi apparatuses, a large filamentous mitochondrion, and an additional small mitochondrion. In Cells D1 and D3, nuclear protrusions were also seen. In all cells, close contact was found between the nucleus and the chloroplast, or between the nucleus and the mitochondrion, or between the mitochondrion and the chloroplast.

To estimate the relationship of these cells, a volumetric analysis was attempted for various cell components of the four *C. applanata* cells (Supplementary Table S2 in Online Resource 1), along with the corresponding average values for *C. reinhardtii*. The cell volume (either within the cell wall or plasma membrane) and the volumes of chloroplast, nucleus, mitochondria, and Golgi apparatuses of *C. applanata* cells A and B were similar to (or slightly smaller than) those of *C. reinhardtii* in the standard condition, in which *C. applanata* cells were also grown. Cell volume doubled in Cell C and Cell D. Accordingly, the four daughter cells were half-volume. The chloroplast and mitochondrial volumes were similar in Cell C and Cell D. These data justify that Cell C represents the stage before cell division. The apparent lack of pyrenoid in Cell C is similar to the dispersion of pyrenoid reported in dividing *C. reinhardtii* cells (Freeman Rosenzweig et al. 2017). A curiously large nuclear volume was obtained for Cell C: the nucleus could enlarge four-fold before the cell division.

We acknowledge that further extensive work is needed to identify the structural changes during the cell cycle of *C. applanata*. However, a significant finding in the present study is that *C. applanata* has a chloroplast with clefts and holes similar to *C. reinhardtii*, although the exact structure was different in the two distantly related algae.

### Additional observation: tight contact between different organelles

The mitochondrion was frequently in tight contact with the chloroplast in *C. applanata* (Supplementary Fig. S4 in Online Resource 1). The mitochondrial membrane (outer membrane) is attached to the outer envelope of the chloroplast over several hundreds of nanometers. The attachment site was often challenging to examine in detail because the section was not always perpendicular to the membranes’ plane. However, the presented figure showed a close contact between the mitochondrial and chloroplast membranes: the two membranes were hardly distinguished in the closest attachment region. Tight contact of mitochondrion and chloroplast was frequently found in *C. applanata* (see Supplementary Fig. S5, S6, and S7 in Online Resource 1). Other types of organelle contacts were also found in these figures.

## Discussion

### Three dimensional structures of two species of *Chlamydomonas* cells

Three dimensional reconstruction of cellular structure with digital data is a powerful tool in cell biology that enables us to view the structure from various directions in different ways. FE-SEM provides cleanly delineated images of cell components, superior to the TEM images and suitable for 3D reconstruction. The resolution of a 3D structure is limited by the thickness of sections, which is currently about 100 nm. Accordingly, the resulting 3D structure is not refined in the direction of depth. Nevertheless, the 3D structures are easy to manipulate by combining selected organelles or cells, comparing different cells, or rotating objects for specific purposes.

The present study was initially intended to construct 3D structures of the chloroplast of *C. reinhardtii*, which was once shown to have a complex structure but was later believed to be cup-shaped. We then try to present a more realistic image of the chloroplast of two strains of *Chlamydomonas*. It is not clear why the chloroplast of *C. reinhardtii* (or “Chlamydomonas”) is currently described as “cup-shaped.” Many cross-section images of electron micrographs or confocal fluorescence micrographs of *C. reinhardtii* make us believe we are observing a cup-like structure. This shape is easy to imagine and to draw in diagrams. However, this is different from the actual chloroplast structure. In both *C. reinhardtii* and *C. applanata*, the 3D shape of the chloroplast is really complex, as we have already described above. Researchers know that cross-section images give partial information on the shape of organelles. However, they do not try 3D reconstruction because it is technically demanding, requires special equipment and software, and is long and tedious work. The semi-automatic image acquisition systems, such as the one used in the present study, will facilitate 3D image processing.

Major findings of the 3D structure construction include not only the complex structure of the chloroplast with openings, clefts, and holes but also the relationship of the chloroplast with other cellular structures, such as the nucleus, the mitochondria, the Golgi apparatuses, and the lipid droplets. The localization of the Golgi apparatus within or near the chloroplast opening or the large chloroplast hole may be favorable for the transport of substances processed by the Golgi to the outer surface of the cell. This transport would not be possible if the chloroplast were literally “cup-shaped.”

### Localization of lipid droplets within or near the chloroplast holes

The localization of the lipid droplets with respect to the chloroplast is closely related to the shape of the chloroplast. The observation explains the previous enigma: some reports argued that lipid droplets were present within the chloroplast stroma (e.g., Goold et al. 2016). This possibility has been excluded by various lines of evidence, including fluorescence microscopy and TEM observation in a previous paper (Moriyama et al. 2018). However, the 3D reconstruction of the cell structure in the present work provides more intuitive evidence that the lipid droplets apparently found within the chloroplast could be explained by their location within the chloroplast holes. The hole could be very large (such as the one seen in Fig. 2b, f, k) to accommodate a large lipid droplet with a diameter as large as 1 µm or more, produced in extreme conditions such as nitrogen deficiency (Moriyama et al. 2018). In some previous studies, the lipid droplets within the chloroplast holes could have been misinterpreted as evidence for the lipid droplets within the chloroplast stroma. The “chloroplast lipid droplets” may have been influenced by the prevailing image of “spheroid chloroplast” with a smooth, convex surface. The researchers might not have suspected large and small holes or clefts in the chloroplast, although Schötz (1972) already showed this by classical 3D reconstruction. The complex shape of the chloroplast, as shown in the present study, could change the view of researchers in various areas of research.

### Generality of complex chloroplast shape

Complex shape is not limited to *Chlamydomonas* chloroplasts. Shalla (1964) has already described: “During periods of most active synthesis of virus, many chloroplasts were distorted and appeared to possess large vacuoles, some of which contained TMV particles or various cytoplasmic organelles such as mitochondria (p. 256).” This was explained as “envelopment of ground cytoplasm by cup-like projections of the plastid was one means by which the vacuoles arose (p. 261).” In a review article, Thomson and Whatley (1980) described that “the amoeboid stage represents the transition of the plastid from a proplastid state to that of a chloroplast (p. 381).” Gielvanowska and Szezuka (2005) described: “The outer chloroplast membrane forms vesicles, and pockets are invaginations of both membranes. The invaginations contain small vesicles, mitochondria, or lipid droplets (Abstract).” Holes and pockets were unambiguously identified by 3D reconstruction in plant chloroplasts (Oi et al. 2017; Yamane et al. 2018). In contrast, a chloroplast stromule is a protruding structure extending from the main body of a chloroplast (e.g., Itoh et al. 2018). Therefore, chloroplast shape is not just spheroid, as illustrated by many researchers since Schimper (1883). We have to renew our image of chloroplast. The 3D structures in the STL format provided as Supplementary Data S1, S2 and S3 (Online Resource 7, 8 and 9) will be helpful for further creating digital illustrations of chloroplast-related topics.

### Mathematical and biological reflections on the chloroplast holes

**Fig. 7:**
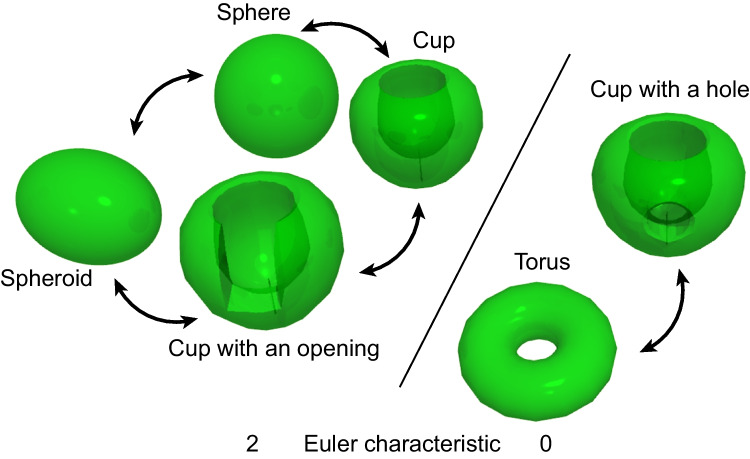
Homeomorphism of closed surfaces. Sphere, spheroid, cup, and cup with an opening are homeomorphic, having the Euler characteristic of 2. Torus and cup with a hole are homeomorphic, having the Euler characteristic of 0. The presence of a hole implies the importance of negative curvature and thus introduces a fundamental difference in both mathematics and biology

The presence of holes or windows in the chloroplast lobe has an essential mathematical problem from the topological point of view because, mathematically, the presence of a hole drastically changes the topological property of a closed surface (Fig. 7). In the light of the homeomorphism of closed surfaces, a sphere and a spheroid are equivalent. Moreover, even a cup, or a cup with an opening, is equivalent to a sphere: they are topologically homeomorphic. However, a cup with a hole is homeomorphic to a torus (or a doughnut with a hole). The two types of closed surfaces are distinguished by the Euler characteristic, which is commonly defined for polygons as well as manifolds. Any closed surface has the Euler characteristic of 2, whereas a closed surface having a hole, such as a torus, has the Euler characteristic of 0. According to the Gauss-Bonnet theorem (see introductory textbooks such as Lee 2011; Muñoz et al. 2020), the Euler characteristic is proportional to the integrated curvature over the entire surface. Lee (2011) concisely explains: “If the surface is topologically equivalent to an *n*-holed doughnut surface, the theorem says that the total curvature is exactly equal to 4π − 4π*n*. In the case *n* = 1 this implies that no matter how a one-holed doughnut surface is bent or stretched, the regions of positive and negative curvature will always precisely cancel each other out so that the total curvature is zero” (p. 9). In other words, even a tiny hole introduces a large negative curvature (concaveness), which is equivalent to the sum of all positive curvature (convexity). In biological words, the closed surface corresponds to the chloroplast envelope membranes. The negative curvature of the envelope membrane must be realized by some mechanism or force exerting on the membrane from the outside or cytoplasm. Indeed, holes are not common in organelles. The exceptionally complex shape, including windows and holes of the chloroplast, must be a product of the action of the cytoplasm, namely, the eukaryotic compartment, if the chloroplast is regarded as prokaryotic. In this regard, the determinant of the chloroplast shape is not just the chloroplast itself. The cytoplasm must be involved in the formation of holes.

### Organelle shape and the endosymbiotic theory

One of the authors (NS) recently cast doubt on the validity of the endosymbiotic origin of chloroplasts, mainly based on phylogenetic analysis of various enzymes located in the chloroplasts (Sato 2020a, 2020b, 2021). The principal evidence for the endosymbiotic or cyanobacterial origin of chloroplasts resides in the phylogenetic and synteny analysis of the ribosomal RNA and various enzymes encoded by the chloroplast genome. However, the original idea of the endosymbiotic theory was inspired by the morphological similarity of chloroplasts and cyanobacteria (then called blue-green algae) (Mereschkowsky 1905; Schimper 1883). The resemblance of the two entities remained helpful in explaining the endosymbiotic theory in many textbooks. The endosymbiotic origin of mitochondria was envisaged after the chloroplast origin (for the history, see Sato 2020b). However, the morphological resemblance between the mitochondria and proteobacteria is no longer valid because of the complex morphology of mitochondria, as shown in the present study. In addition, the origin of about 90% of the mitochondrial enzymes is known to be older than the mitochondrial genome (Gray 2015; Pittis and Gabaldón 2016). The evidence for the endosymbiotic origin of mitochondria became limited to the phylogenetic analysis of the genes encoded by the mitochondrial genome. The situation in chloroplast seems more favorable for the endosymbiotic origin, mainly because the morphological similarity of chloroplasts and cyanobacteria is easy to understand for non-specialists unfamiliar with the details of phylogenetic analysis. We now have ample data that the chloroplasts are not spheroid, like mitochondria. They are no longer similar to any cyanobacteria. We must re-examine the evidence that supports the endosymbiotic origin of chloroplasts and mitochondria. The topological considerations (previous section) also suggest that the shape of the chloroplast envelope membranes are the product of both cytoplasm and chloroplast. The present study will contribute to such re-examination.

## Supplementary Information

Below is the link to the electronic supplementary material.

Supplementary file1 (PDF 32.4 MB)

Supplementary file2 (MP4 4211 KB)

Supplementary file3 (MP4 1780 KB)

Supplementary file4 (MP4 12233 KB)

Supplementary file5 (MP4 1169 KB)

Supplementary file6 (MP4 3882 KB)

Supplementary file7 (ZIP 9.70 MB)

Supplementary file8 (ZIP 16.5 MB)

Supplementary file9 (ZIP 23.9 MB)
