# Supplementary material for: Chloroplasts with clefts and holes: a reassessment of the chloroplast shape using 3D FE-SEM cellular reconstruction of two species of Chlamydomonas
Source: Protoplasma. 2024 Sep 23;262(1):207–18. doi: 10.1007/s00709-024-01990-7 (PMC11698768; doi:10.1007/s00709-024-01990-7)
Supplement: Supplementary file 1 — Supplementary file1 (PDF 32.4 MB) [file 709_2024_1990_MOESM1_ESM.pdf]

Online Resources

Article title: **Chloroplasts with clefts and holes: a reassessment of the chloroplast shape using 3D FE-SEM cellular reconstruction of two species of *Chlamydomonas***

Journal name: Protoplasma

Authors: Naoki Sato*, Mayuko Sato, Mayumi Wakazaki, Takashi Moriyama, Takashi Hirashima, and Kiminori Toyooka

Corresponding author: Naoki Sato
Graduate School of Arts and Sciences, The University of Tokyo, Komaba, Meguro-ku, Tokyo, 153-8902 Japan
naokisat@bio.c.u-tokyo.ac.jp, naokisat@green.ocn.ne.jp

Online Resource 1 (this file)

Table S1 Chloroplast dimension in µm.

Table S2 Volumetry of cellular components in *C. applanata* and *C. reinhardtii.*

Fig. S1 Various views of a *C. reinhardtii* cell (Cell 2) grown in the standard condition.

Fig. S2 Serial images of lipid droplets in *C. reinhardtii* grown in the lipid-accumulating condition.

Fig. S3 Lipid droplets in chloroplast holes in *C. reinhardtii* grown in the lipid-accumulating condition.

Fig. S4 Close contact between mitochondrion and chloroplast.

Fig. S5 High-resolution serial images showing the curious mitochondrial morphology in a *C. applanata* cell.

Fig. S6 High-resolution serial images showing the curious mitochondrial morphology in another *C. applanata* cell.

Fig. S7 High-resolution serial images showing the curious mitochondrial morphology in another *C. applanata* cell.

Online Resource 2 (MP4) Movie S1 Rotating view of the chloroplast of *C. reinhardtii* Cell 2 grown in the standard condition. This movie was prepared by the Shade 3D software. Flagella, lipid droplets, and Golgi apparatuses are also shown. The movie flows relatively fast but can be stopped at any point using the cursor and manually manipulated to go back and forth, so it is possible to examine the structure in detail. This is true for all movies.

Online Resource 3 (MP4) Movie S2 Rotating view of the chloroplast (with flagella) of *C. reinhardtii* cells grown in the standard condition.

Online Resource 4 (MP4) Movie S3 Rotating view of the chloroplast (with flagella) of *C. reinhardtii* cells grown in the lipid-accumulating, high-light condition.

Online Resource 5 (MP4) Movie S4 Rotating view of the chloroplasts of *C. reinhardtii* and *C. applanata*. In the starting scene: left, *C. reinhardtii* chloroplast in the standard condition; center, *C. reinhardtii* chloroplast in the lipid-accumulating condition; right, *C. applanata* chloroplast (Cell A in Fig. 6). Chloroplasts are shown as half-transparent object. Eye spot (dark purple) and pyrenoid (brown) are also shown. Flagella (purple) are shown

to identify the orientation of chloroplast. Pyrenoid is located at the posterior end of the *C. reinhardtii* chloroplasts but in the middle of the *C. applanata* chloroplast. Note that no eye spot was detected in this particular *C. applanata* chloroplast.

Online Resource 6 (MP4) Movie S5 Rotating view of *C. applanata* chloroplasts with flagella, Golgi apparatuses and lipid droplets.

Online Resource 7 (ZIP) Data S1 STL data for *C. reinhardtii* cells in the standard condition
Zip-archived 3D surface model files. After unzipping, there is a folder containing data for the cells. For each cell, STL models of cell components are presented. Each STL model was simplified by remeshing to reduce file size. STL files can be viewed with any 3D viewer software.

Online Resource 8 (ZIP) Data S2 STL data for *C. reinhardtii* cells in the lipid-accumulating condition

Online Resource 9 (ZIP) Data S3 STL data for *C. applanata* cells

Table S1 Chloroplast dimensions in μm

	Length	Width	Height
C. reinhardtii standard			
Cell 1	7.58	6.06	5.79
Cell 2	7.40	6.19	6.07
Cell 3	8.91	5.19	5.11
C. reinhardtii lipid-accumulating			
Cell 1	7.57	6.93	7.23
Cell 2	9.06	6.33	6.99
Cell 3	8.67	7.50	6.17
Cell 4	7.83	7.20	7.02
Cell 5	6.49	7.83	7.07
C. applanata			
Cell A	7.38	4.69	3.74
Cell B	5.44	5.37	5.37
Cell C	8.28	5.72	5.04
Cell D1	5.19	3.59	2.93
Cell D2	5.64	2.90	2.88
Cell D3	6.14	3.21	2.87
Cell D4	7.13	2.83	2.41

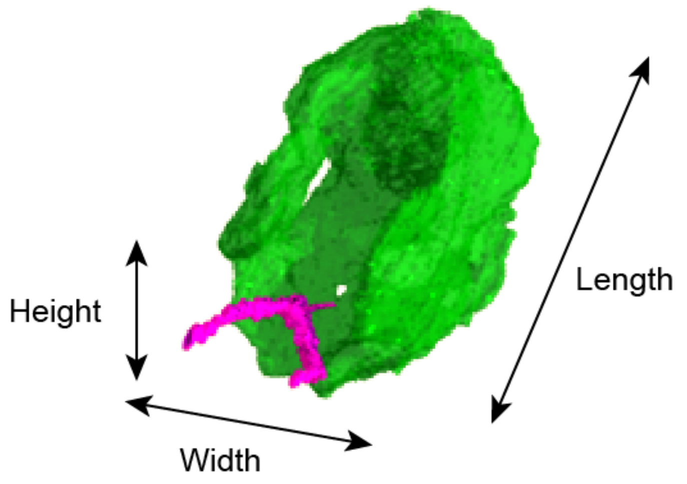

Table S2 Volumetry of cellular components in *C. applanata* and *C. reinhardtii*
The internal volume of the membrane (or wall)-surrounded compartments is shown in μm^3. The metrics of the individual daughter cells of the dividing cell D are also shown. For comparison, the corresponding average volumes for *C. reinhardtii* cells are presented.

	Cell mass including cell wall	Cell mass	Chloro-plast	Vacuoles	Nucleus	Mito-chondria	Lipid droplets	Golgi
C. applanata								
Cell A	84.9	79.2	38.2	6.28	4.07	1.69	0.41	0.17
Cell B	95.3	83.4	44.7	7.11	4.80	1.49	0.63	0.20
Cell C	154.0	141.7	59.2	4.50	13.92	2.60	0.43	0.58
Cell D total	124.2	113.8	58.1	5.05	8.45	2.87	0.49	0.42
Cell D1	33.4	38.1	15.6	1.27	2.26	0.81	0.15	0.12
Cell D2	30.8	18.1	14.6	1.30	2.00	0.69	0.09	0.10
Cell D3	28.3	26.0	14.1	1.25	2.07	0.59	0.12	0.10
Cell D4	31.6	31.6	13.9	1.23	2.12	0.86	0.13	0.10
C. reinhardtii								
Standard	85.7	76.5	31.8	9.26	4.95	1.65	0.45	0.26
Lipid-accu mulating	122.7	109.3	58.9	8.20	4.66	1.56	1.07	0.14

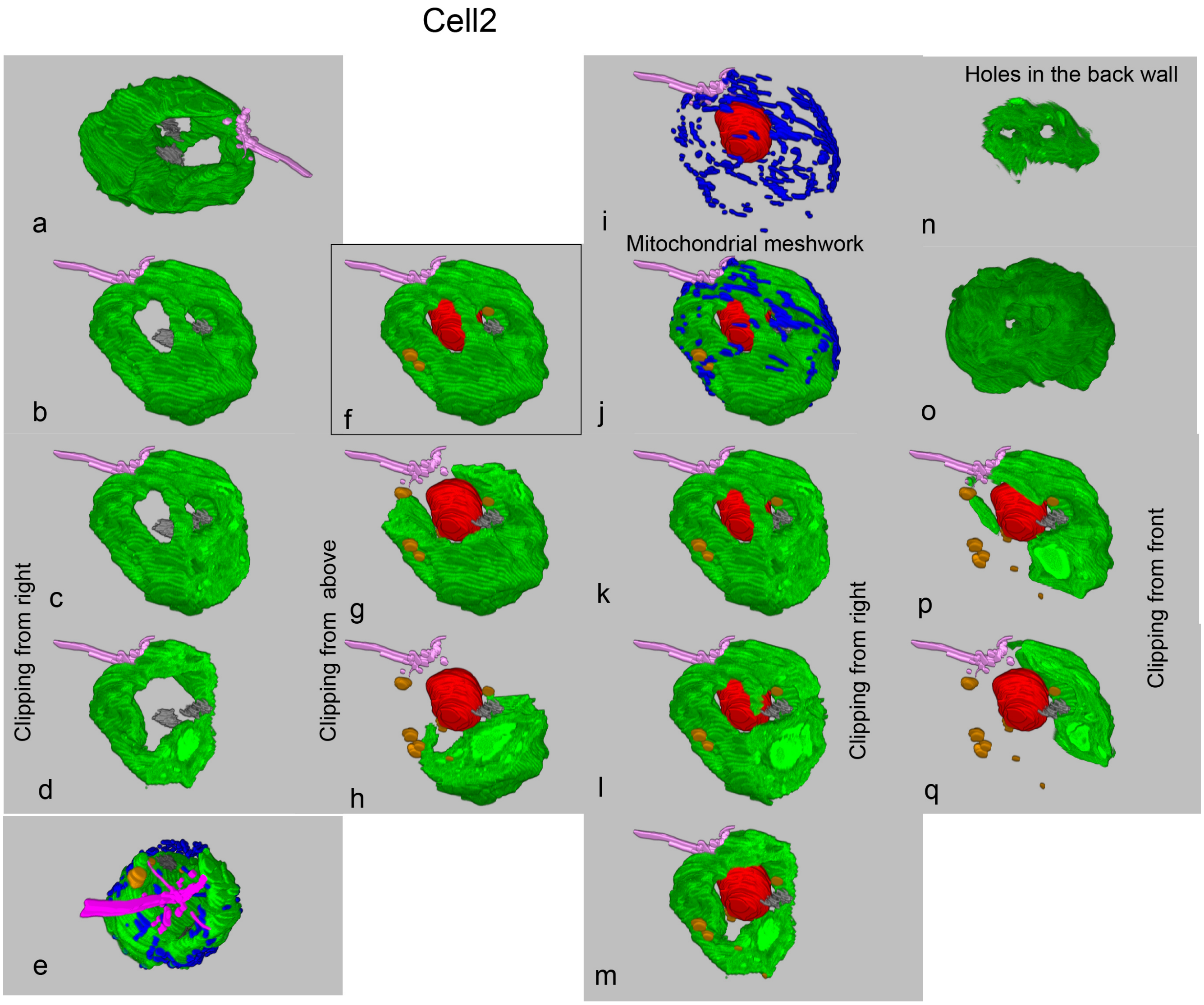

Fig. S1 Various views of a *C. reinhardtii* cell (Cell 2) grown in the standard condition
Panel f is identical to Panel g of Fig. 2. Other panels show different views with or without nucleus, view with clipping from the right, top, or front. (e) Whole cell view from the anterior end. (i) Mitochondrial meshwork. (n) Part of the chloroplast lobe with small holes.

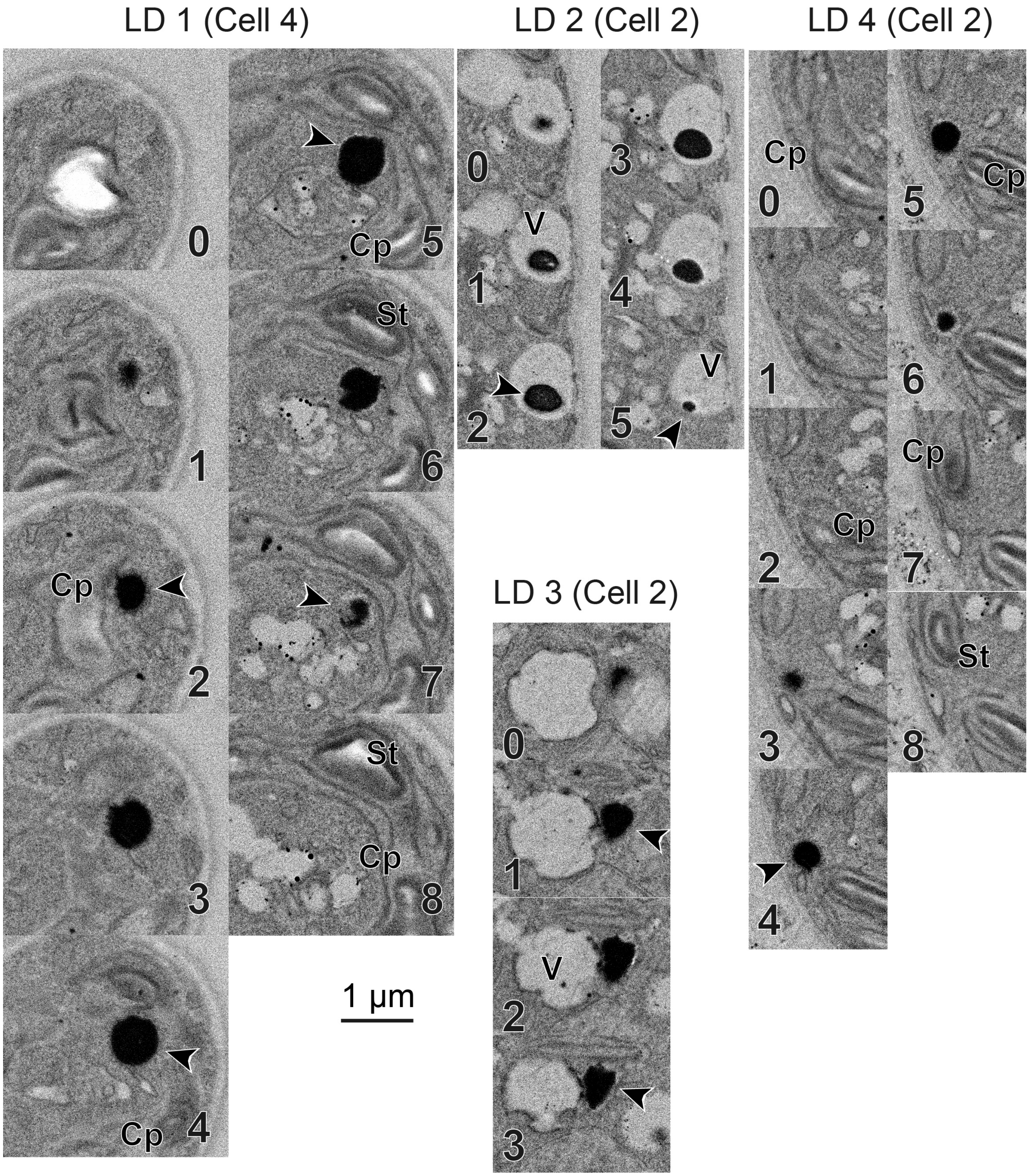

Fig. S2 Serial images of lipid droplets in *C. reinhardtii* grown in the lipid-accumulating condition
Four sets of serial images of lipid droplets (LD) are shown. Each arrowhead indicates a lipid droplet. Cp, chloroplast; St, starch granule; V, vacuole. Note that each of LD1 and LD4 was present within an opening of chloroplast. LD2 and LD3 were in close contact with a vacuole.

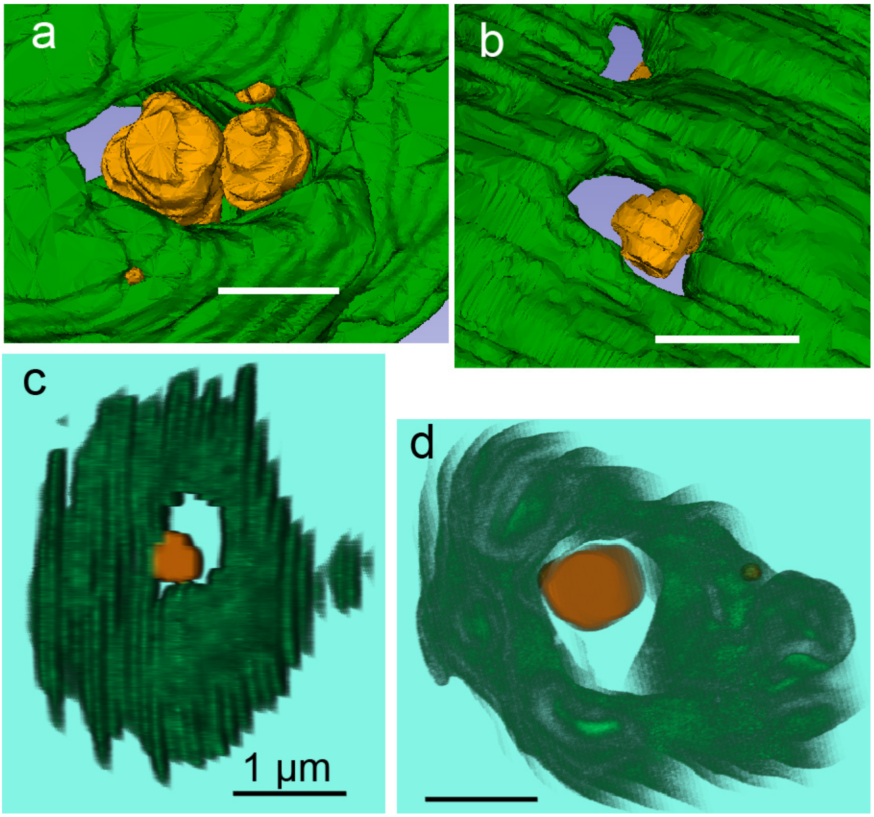

Fig. S3 Lipid droplets in chloroplast holes in *C. reinhardtii* grown in the lipid-accumulating condition.. **a** and **b**, perspective view prepared by 3D Slicer, **c** and **d**, cross-section prepared by Fluorender. **a**, Cell 1; **b** and **d**, Cell 2 (LD4 in **Fig. S2**); **c**, Cell 4 (LD1 in **Fig. S2**). Green, chloroplast; orange or brown, lipid droplet.

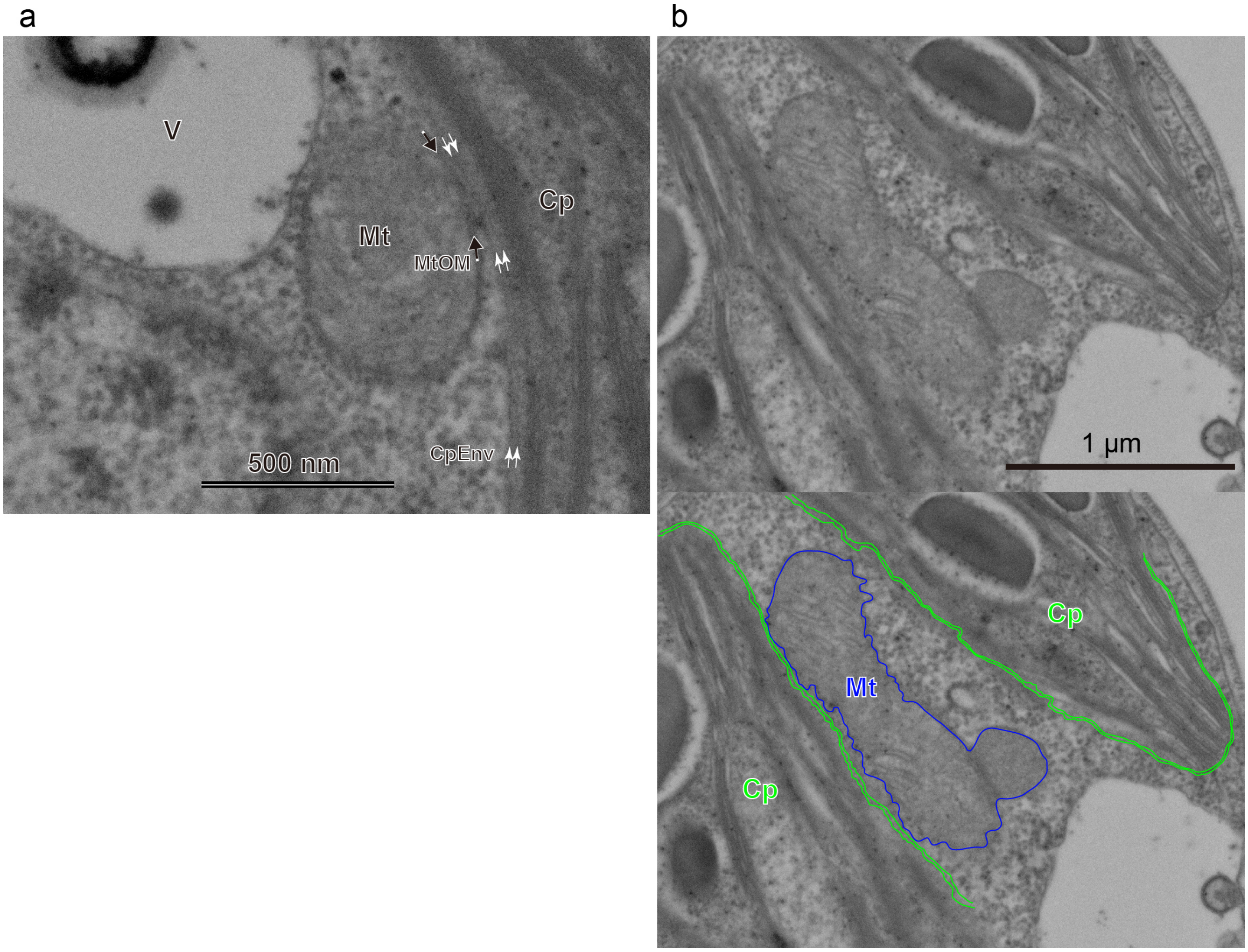

Fig. S4 Close contact between mitochondrion and chloroplast in *C. applanata* cells
Cp, chloroplast; Mt, mitochondrion. In (a), the chloroplast envelope membranes are marked with two white arrows, whereas the mitochondrial outer membrane is indicated by black arrows. Tight attachment is found between the two arrow sets (middle of the characters "Cp" and "Mt").
Panel (b) is shown in two ways: the top panel is the original image, whereas the bottom image is annotated to show the membranes. Tight attachment is found over a long region.

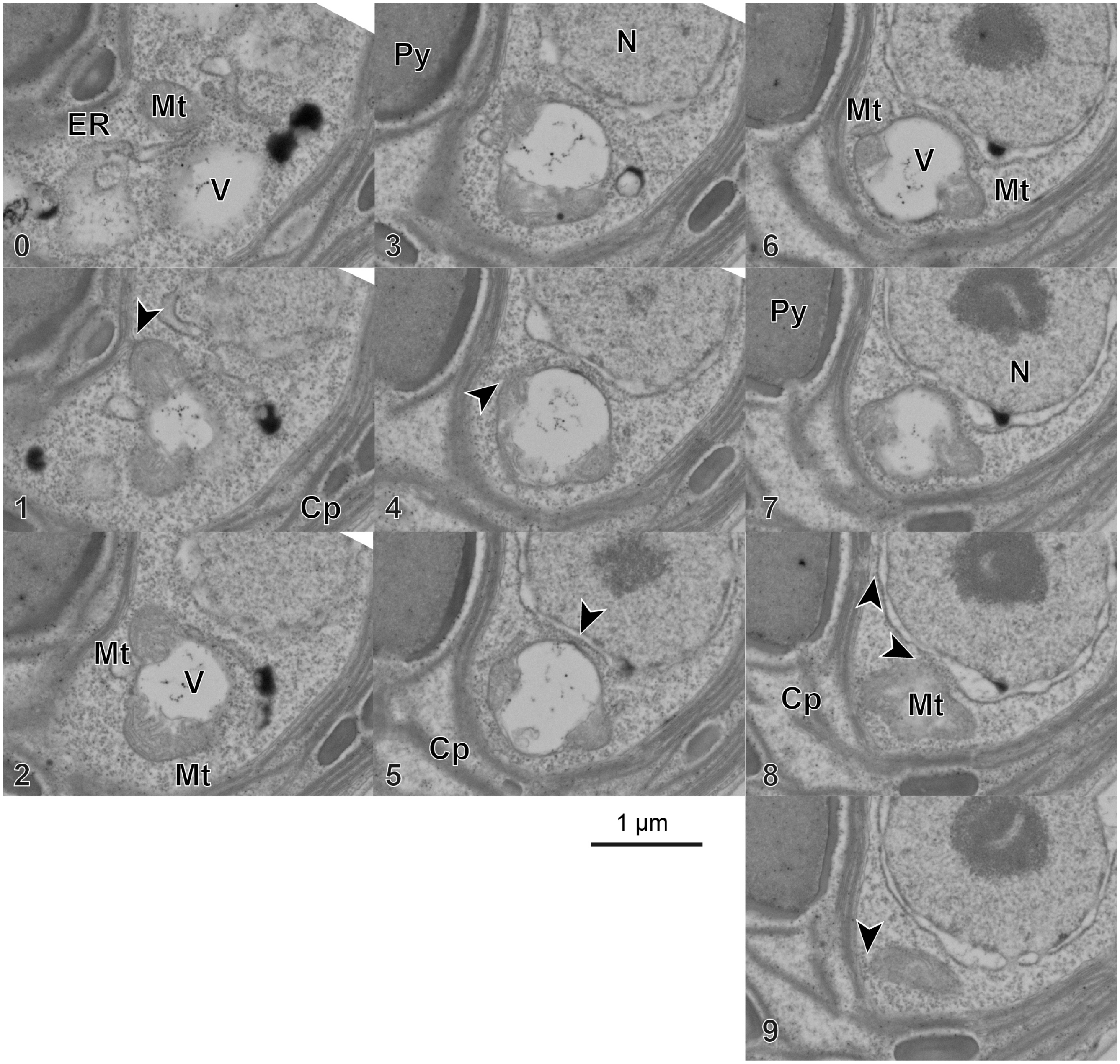

Fig. S5 High-resolution serial images showing the curious mitochondrial morphology in a *C. applanata* cell. Cp, chloroplast; Mt, mitochondrion; N, nucleus; Py, pyrenoid; V, vacuole. Each arrowhead points to an organellar contact site.

We often noted that a mitochondrion contacted other organelles in *C. applanata*: nuclear protrusion into mitochondrion was pointed out in the text. Another intriguing type of contact was the vacuolar intrusion into the mitochondrion (Supplementary Fig. S5, S65, and S7). At first glance, it appeared as a hollow space in the mitochondrion. It could have been a fixation artifact, but a close examination of serial sections revealed that a vacuole intruded into a mitochondrion. There was a vacuolar limiting membrane and the mitochondrial outer membrane at the interface of the vacuolated space and the mitochondrial matrix. The vacuolated space was not an unstructured or broken part of the mitochondrion. The vacuolar intrusion was often observed at the branching point of the mitochondrion, resulting in an image of two or three mitochondria attached to a vacuole. Contact of mitochondrion and nucleus or contact of chloroplast and nucleus were also seen in these figures.

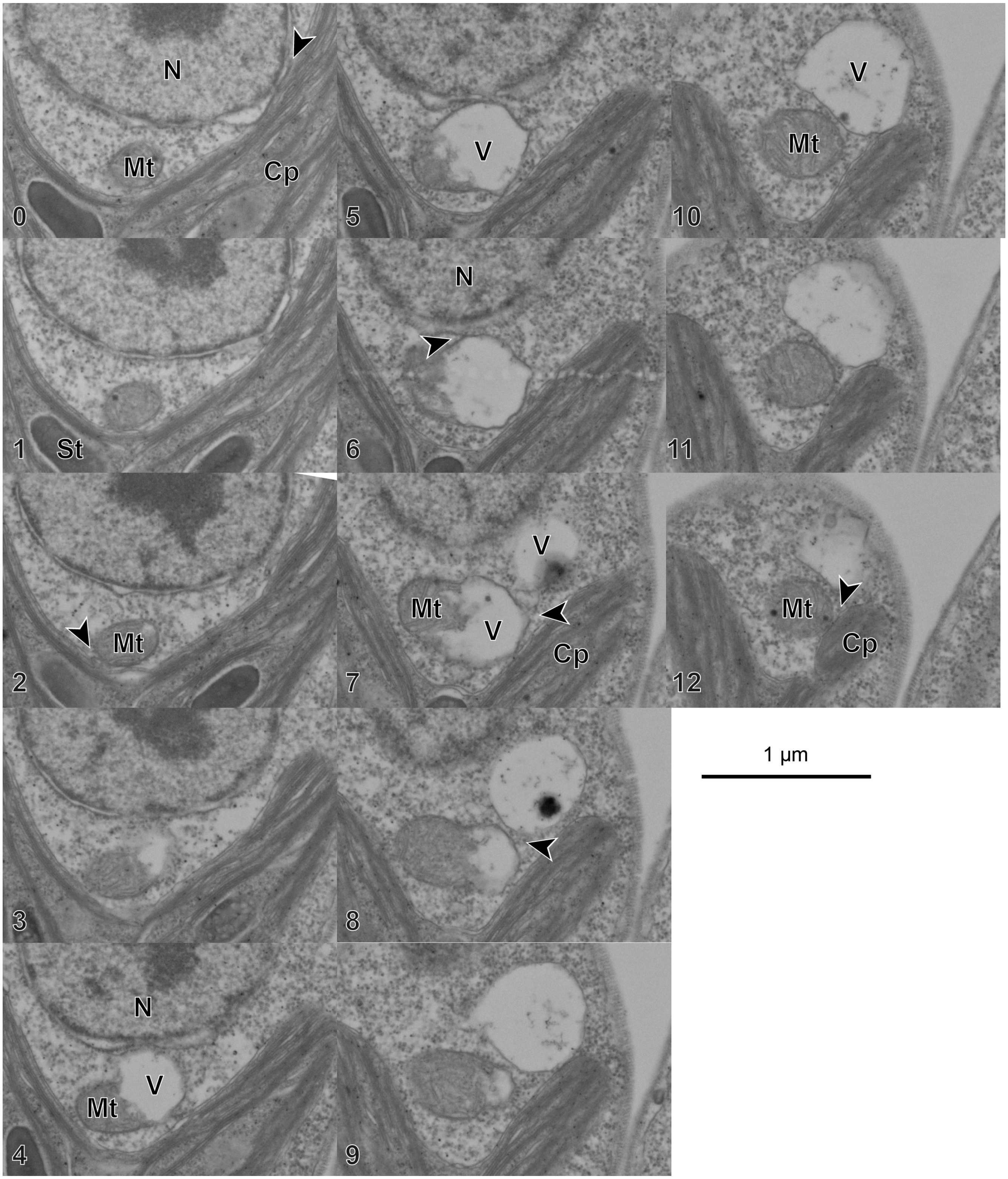

Fig. S6 High-resolution serial images showing the curious mitochondrial morphology in another *C. applanata* cell.

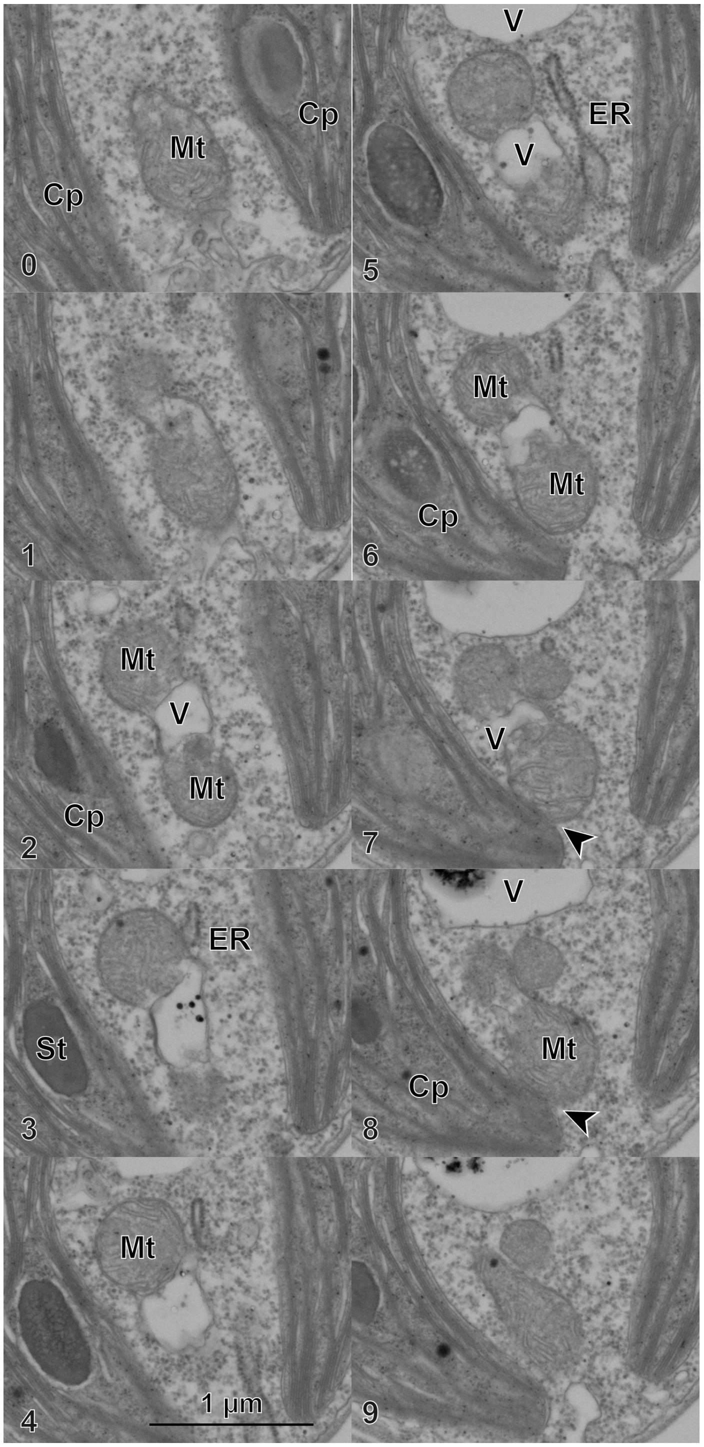

Fig. S7 High-resolution serial images showing the curious mitochondrial morphology in another *C. applanata* cell. ER, endoplasmic reticulum.
